# Special Issue on Nanoscale Thermodynamics

**DOI:** 10.3390/nano11030584

**Published:** 2021-02-26

**Authors:** Signe Kjelstrup

**Affiliations:** PoreLab, Department of Chemistry, Faculty of Natural Sciences, Norwegian University of Science and Technology, 7491 Trondheim, Norway; signe.kjelstrup@ntnu.no

This Special Issue concerns recent developments of a theory for energy conversion on the nanoscale, namely nanothermodynamics. The theory applies to porous media, small surfaces, clusters or fluids under confinement. There are a large number of unsolved issues in these contexts and present efforts only paint part of the broader picture. We may still ask questions on how far down in scale we can really use the Gibbs equation. Which theory can replace the Gibbs equation beyond the thermodynamic limit? 

It is well known that confinement can change the equation of the state of a fluid, but how does confinement change the equilibrium conditions? How do we formulate equilibrium conditions on the nanoscale, and what are the independent variables? To deal with equilibrium alone seems a formidable task, let alone how to extend the descriptions to systems away from equilibrium.

This Special Issue explores in more detail some roads that were opened by Hill when he launched his thermodynamics for small systems in 1963. His method has, however, not gained much attention since it was published. We now consider this an underused opportunity. The theoretical developments in nanothechnology need to follow the experimental progress, and that is rapid. It is our ambition, therefore, to aspire to an increased effort that can further develop suitable theoretical tools and methods in nanoscience. All ten contributions to this Special Issue can be seen as efforts to support, enhance and validate such theoretical developments.

The first two papers [1,2] demonstrate the use of Hill’s nanothermodynamics in new settings. The Small System Method for the determination of thermodynamic factors has already been successfully applied in many contexts. The method exploits the small system’s scaling properties or size dependencies. In this Special Issue, Dawass et al. [1] show how the analysis of Kirkwood Buff integrals can be made more accurate. They conclude that this is possible, by applying three methods to compute these integrals in the thermodynamic limit. Radial distribution functions (RDFs) of finite systems are used. Tripathy et al. [2] extend the Small System Method further to also characterize the hydration shell compressibility of a generally hydrophobic polymer in water. They show how this finding may be generalized to study hydrophobic interactions.

The next three papers [3,4,5] concern the pressure of confined fluids and their description in equilibrium. Rauter et al. show [3] that the integral pressure is constant across phase boundaries and that this finding is equivalent to assuming validity of Young’s and Young–Laplace’s law. In agreement with this, Máté Erdős et al. [4] document the inter-relation of the differential and the integral pressure. Galteland et al. [5] show how the disjoining pressure can be understood using Hill’s theory, and present Maxwell relations for small systems. 

The issue contains three extensions of Hill’s theory, see papers [6,7,8]. Hill stated that small systems do not obey Legendre transforms, a clear disadvantage. Beering et al. [6] have been able to show for the first time in their article “*A Legendre–Fenchel Transform for Molecular Stretching Energies*” that an alternative for small systems lies in the Legendre–Fenchel transform. This is an extension of Hill’s theory that may prove useful in practice. Strøm et al. [7] have been able to extend on Hill’s examples, by considering adsorbed films on very small clusters. Their article “*When Thermodynamic Properties of Adsorbent Films Depends on Size*” offers a new way to deal with adsorption on, say, atmospheric particles. In yet another article, Strøm et al. [8] were able to compute and illustrate the equation of state of an ideal gas when it becomes confined. Large discrepancies from normal ideal gas behavior are found. The equation of state can be computed exactly for an ideal gas using statistical mechanics and is illustrated by molecular dynamics simulations.

The last two papers [9,10] are special. The only experimental paper in this issue is provided by Men’shikov et al. [9]. The authors show how sensitive the structure is to environmental conditions, and how the results for adsorption enthalpy vary with temperature, carbon porosity and surface area. Therefore, there are clear indications of multilayer formations on some of their activated carbons, and abnormal effects, which may benefit from other theories. So far, the treatment of these data has followed the classical scheme. Is there an alternative route, simpler than that of Hill, to nanothermodynamics? Rodrigo de Miguel and J. Miguel Rubi [10] propose this in their study on “*Statistical Mechanics at Strong Coupling: A Bridge between Landsberg’s Energy Levels and Hill’s Nanothermodynamics*”. They review and show the connection between three theories, including Hill’s, proposed for the thermodynamic treatment of systems that do not obey the additivity ansatz of classical thermodynamics.
